# Local and Systemic Immune Responses to Influenza A Virus Infection in Pneumonia and Encephalitis Mouse Models

**DOI:** 10.1155/2017/2594231

**Published:** 2017-08-24

**Authors:** Yoshiharu Nagaoka, Nobuyuki Nosaka, Mutsuko Yamada, Masato Yashiro, Yosuke Washio, Kenji Baba, Tsuneo Morishima, Hirokazu Tsukahara

**Affiliations:** Department of Pediatrics, Okayama University Graduate School of Medicine, Dentistry and Pharmaceutical Sciences, Okayama, Japan

## Abstract

**Objective:**

To compare local and systemic profiles between different disease pathologies (pneumonia and encephalitis) induced by influenza A virus (IAV).

**Methods:**

An IAV pneumonia model was created by intranasal inoculation of C57BL/6 mice with influenza A/WSN/33 (H1N1) virus. Lung lavage and blood collection were performed on day 3 after IAV inoculation. Similarly, an IAV encephalitis mouse model was created by direct intracranial IAV inoculation. Cerebrospinal fluid (CSF) and blood collection were conducted according to the same schedule. Cytokine/chemokine profiles were produced for each collected sample. Then the data were compared visually using radar charts.

**Results:**

Serum cytokine profiles were similar in pneumonia and encephalitis models, but local responses between the bronchoalveolar lavage fluid (BALF) in the pneumonia model and CSF in the encephalitis model differed. Moreover, to varying degrees, the profiles of local cytokines/chemokines differed from those of serum in both the pneumonia and encephalitis models.

**Conclusion:**

Investigating local samples such as BALF and CSF is important for evaluating local immune responses, providing insight into pathology at the primary loci of infection. Serum data alone might be insufficient to elucidate local immune responses and might not enable clinicians to devise the most appropriate treatment strategies.

## 1. Introduction

Cytokines and chemokines are key factors in the pathogenesis of influenza A virus (IAV) infection in human and animal models. Numerous studies of various IAV strains have revealed the involvement of various cytokines/chemokines in the pathology of this organism [1]. IAV infection causes various diseases in different organs, ranging from pneumonia [2–7] to encephalitis/encephalopathy [8–11]. To devise appropriate treatment strategies, the pathological events occurring at the primary loci of disease must be elucidated. Most reports describe studies that have used serum to investigate the cytokine/chemokine profiles of IAV-infected patients, especially pneumonia patients [2–7]. Nevertheless, it remains unclear whether these serum data correlate with the local immune response against IAV.

This background has prompted us to characterize the inflammatory mediator response in serum compared with local samples, that is, bronchoalveolar lavage fluid (BALF) and cerebrospinal fluid (CSF) in IAV pneumonia and IAV encephalitis mouse models, respectively. This report presents evidence that interpreting a serum cytokine/chemokine profile in terms of a local immune response against IAV infection can be misleading.

## 2. Materials and Methods

### 2.1. Ethics

The Animal Use Committee of the Okayama University Graduate School of Medicine, Dentistry and Pharmaceutical Sciences approved this study (number OKU-2012628), which was conducted in accordance with the National Institutes of Health Guidelines.

### 2.2. Experimental Animals

Eight-week-old male C57BL/6 mice were purchased from Charles River Laboratories Japan Inc. (Yokohama, Japan). The mice were housed in a specific pathogen-free animal facility at 25°C with a 12 hr light/dark cycle. They were fed a standard diet (Oriental MF; Oriental Yeast Co. Ltd., Tokyo, Japan).

### 2.3. Preparation of Viral Inocula

Influenza A/WSN/33 (H1N1) virus was kindly provided by the Department of Microbiology, Kawasaki Medical University. This mouse-adapted H1N1 human IAV strain is not only pneumotropic after intranasal inoculation; it is also neurovirulent after inoculation into the CSF [12, 13]. We used this strain throughout this study. The virus was grown in 10-day-old embryonated chicken eggs. The virus titre was quantitated using a plaque assay with Madin-Darby canine kidney cells.

### 2.4. Experimental Processes

Nine-week-old C57BL/6 mice were divided into two groups to create a pneumonia model and an encephalitis model. To create the pneumonia model, the mice were inoculated intranasally with IAV (5.0 × 10^3^ pfu) suspended in 25 *μ*L sterile phosphate-buffered saline (PBS) after being anesthetized with isoflurane. Similarly, to create an encephalitis model, IAV was inoculated directly into the CSF (2.0 × 10^3^ pfu) of the mice. For inoculation of IAV into the CSF, a hole was made in the skull using a 30-gauge (0.9 mm) injection needle. Then the virus was injected slowly in a volume of 25 *μ*L [14]. These IAV doses were selected because they were 100% lethal for each type of infection. Moreover, inoculation to a specific site did not cause infections at other sites. Control mice in each model were inoculated with PBS alone. All mice recovered from the operation. They were examined as described below. The day of virus inoculation was defined as day 0. Body weight of mice infected was monitored for 5 days after inoculation in each model.

Sample collection was performed on day 3 after IAV inoculation (six mice per group at each time point). For the pneumonia model, the mice were euthanized humanely; blood was sampled for cytokine/chemokine measurements. The left lung hilus was subsequently ligated; the right lung was lavaged twice with 500 *μ*L cold PBS through a 20-gauge cannula. The recovered BALF was collected and centrifuged. Then the supernatant was stored at −80°C before cytokine/chemokine analysis. The left lung was preserved for histological analysis (described below). For the encephalitis model, the mice were euthanized humanely. Their blood was sampled. The CSF was collected using the following method. After exteriorizing the foramen magnum with the neck anteflexed with the operator's first finger and thumb, the foramen magnum was punctured with the needle-like thermoformed MICROCAPS (64 mm length, 0.9017 mm O.D., 0.62992 mm I.D.; Drummond Scientific Co., Broomall, PA, USA); CSF was collected using capillarity. Approximately 5 *μ*L of CSF was sampled using this method. Blood-contaminated CSF was removed. Then the collected CSF was stored at −80°C before cytokine/chemokine analysis. Subsequently, the brain was excited and preserved for histological analysis.

Cytokines and chemokines in serum, BALF, and CF including granulocyte-colony stimulating factor (G-CSF), interferon-gamma (IFN-*γ*), interleukin- (IL-) 1*β*, IL-6, IL-10, IL-12, IL-13, IL-15, monocyte chemotactic protein- (MCP-) 1, macrophage inflammatory protein- (MIP-) 1*α*, interferon-gamma-inducible protein- (IP-) 10, and tumor necrosis factor- (TNF-) *α*, were measured using a mouse cytokine/chemokine-magnetic bead panel (Millipore Corp., Billerica, MA, USA) in a Luminex 100 system (Millipore Corp.).

For the evaluation of viral propagation, the lung and brain were harvested immediately after inoculation and on days 1, 3, and 5 after IAV inoculation. The lower half of the left lung and the right anterior portion of the brain were excised and soaked in RNAlator for virus quantification analysis. Total RNA was extracted from the preserved specimens in RNAlator using the RNeasy Plus Mini kit (Qiagen Inc., Hilden, Germany). Total RNA was reverse-transcribed to cDNA using RETROscript (Applied Biosystems, Foster City, CA, USA) according to the manufacturer's instructions. The cDNA was used as a template for PCR (7500 Real-Time PCR System; Applied Biosystems). The probe and primers for the analysis of the expression of influenza virus type A (M gene) mRNA were the following [15]: TaqMan probe, 5′-6CCCTCAAAGCCGAGATCGCACAGAGAC-3′; forward primer, 5′-CGTTCTCTCTATCATCCCGTCAG-3′; reverse primer, 5′-GGTCTTGTCTTTAGCCATTCCATG-3′ [GenBank NC_002016].

The remaining portions of the lung and brain specimens were frozen in optimal cutting temperature compound and were subsequently sliced into 4-*μ*m-thick sections using a cryostat. The cryosections were blocked by acetone. Fluorescent immunohistochemical analysis for IAV nucleoprotein was performed using the anti-influenza A virus nucleoprotein antibody (Abcam plc., Cambridge, UK) according to the manufacturer's instructions.

### 2.5. Statistical Analysis

Comparisons were performed using the Mann–Whitney *U* test with software (Prism 6.0; GraphPad Software Inc., San Diego, CA, USA). A *p* value of *p* < 0.05 was considered statistically significant.

## 3. Results

### 3.1. Confirmation of Infection at Respective Sites

**Figure 1:**
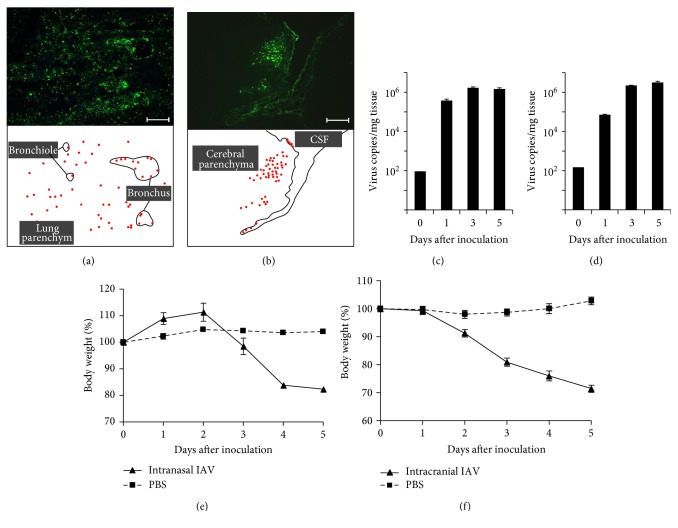
Immunofluorescent staining and virus propagation at each infected site. Immunofluorescent staining of the lung (a) and the brain (b) 5 days postinfection detected influenza A virus (IAV), in the bronchial epithelium and the cerebral cortex, respectively. Images are representative of 4 mice per model. IAV propagation was detected both in the lung (c) and in the brain (d) by reverse transcription polymerase chain reaction (*n* = 3 mice for day 0, *n* = 5–7 mice for days 1, 3, and 5 per group). Body weight of mice infected with intranasal IAV (e) and intracranial IAV (f) was monitored for 5 days after inoculation (*n* = 15 mice for days 0 and 1, *n* = 10 mice for days 2 and 3, and *n* = 5 mice for days 4 and 5 per group). Mice in the pneumonia group lost about 20% of their body weight, whereas mice in the encephalitis group showed about 30% weight loss. All data represent the mean ± SEM values.

Fluorescent immunostaining of the lung obtained from the pneumonia model revealed IAV in the bronchial epithelium (Figure 1(a)). In the encephalitis model, the viral antigen was detected in the third and fourth brain ventricles and in the brain cortex (Figure 1(b)). No viral antigen was detected in the brain in the pneumonia model or in the lung in the encephalitis model (data not shown).

Virus quantification in the infected organs using real-time RT-PCR demonstrated a gradual increase from 10^2^ copies/mg tissue to 10^6^ copies/mg tissue for the first three days, with virus numbers then reaching a plateau in each group (Figures 1(c) and 1(d)). No viral RNA was detected in the brain in the pneumonia group or in the lung in the encephalitis group.

### 3.2. Measurement of Cytokines and Chemokines

**Figure 2:**
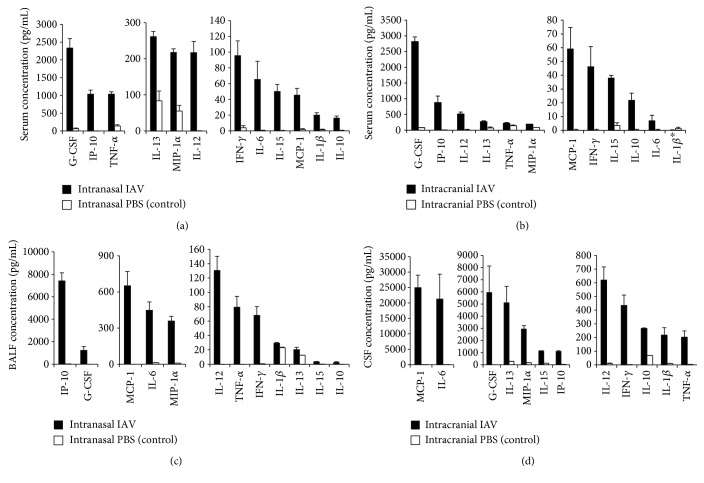
Concentrations of 12 cytokines/chemokines in serum and local samples from pneumonia and encephalitis models. Serum concentrations of cytokines/chemokines in the pneumonia model (a) and the encephalitis model (b). Local concentrations of cytokines/chemokines in the BALF in the pneumonia model (c) and the CSF in the encephalitis model (d). Levels of 12 cytokines/chemokines were measured using a multiplex bead-based assay. Data represent the mean ± SEM values. All samples showed a significant increase in cytokine/chemokine levels for the IAV-infected groups, with the exception of serum IL-1*β* (^∗^) in the encephalitis model (*n* = 6 per group). Statistical comparisons were conducted using the Mann–Whitney *U* test.

Serum cytokines and chemokines were measured to assess the systemic immune response against IAV infection in each model. All 12 cytokines/chemokines investigated were increased significantly in the IAV pneumonia model compared with the control (Figure 2(a)), whereas all factors other than IL-1*β* increased significantly in the IAV encephalitis model compared with the control (Figure 2(b)). The cytokines and chemokines were then measured in the BALF and CSF in the pneumonia and encephalitis models, respectively, to observe local inflammatory responses at the site of infection. All 12 factors were increased significantly in both models compared with the controls (Figures 2(c) and 2(d)).

### 3.3. Comparison of Cytokine/Chemokine Profiles

**Figure 3:**
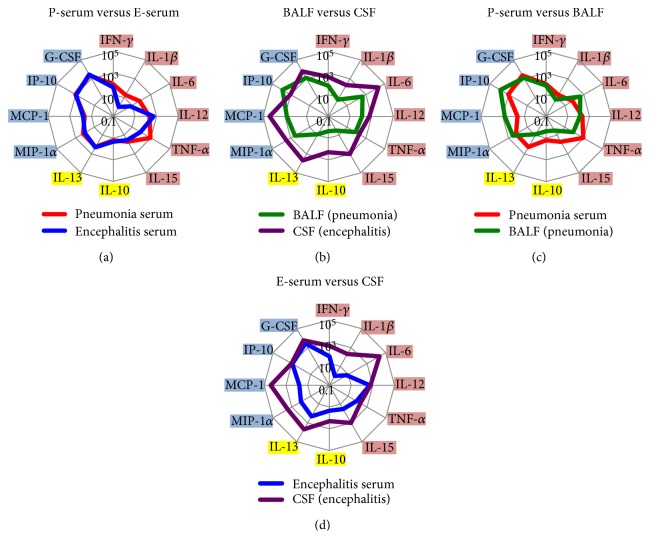
Comparison of cytokine/chemokine profiles as shown by radar charts. Average increase from the basal line of each cytokine/chemokine is shown on a chart with a logarithmic axis. Proinflammatory cytokines (right side of charts), anti-inflammatory cytokines (lower side of charts), and chemokines (left side of charts) are highlighted in pink, yellow, and blue, respectively: P-serum, serum from pneumonia model; E-serum, serum from encephalitis model; BALF, bronchoalveolar lavage fluid from pneumonia model; and CSF, cerebrospinal fluid from encephalitis model.

Because many cytokines and chemokines are involved in the pathogenesis of disease induced by IAV, we inferred that understanding the overall cytokine/chemokine profiles is beneficial for understanding disease pathology. Consequently, we attempted to represent and compare each cytokine/chemokine profile with a radar chart and evaluate the correlation visually (Figure 3). We calculated the average increase from the basal line for each cytokine and chemokine in the IAV-infected models and presented them on charts with a logarithmic axis. On evaluation, we noted that BALF was obtained using PBS. Therefore, relative values rather than absolute values (i.e., shape similarity among radar charts) were important in evaluating the correlation between profiles, especially when comparing the profile of BALF.

Although the serum cytokine/chemokine profiles showed a considerable degree of similarity between both the pneumonia and encephalitis models (Figure 3(a)), the local cytokine/chemokine responses differed between BALF and CSF (Figure 3(b)). In the pneumonia model, the profiles were similar between serum and BALF, but some deviations were identified: a larger shift to the lower side in the serum chart and a larger shift to left side in the BALF chart (Figure 3(c)). In the encephalitis model, a low level of similarity between the profiles for serum and CSF was detected, with the CSF chart showing a larger overall increase than the serum chart (Figure 3(d)).

## 4. Discussion

Cytokines and chemokines contribute to the overall pathology of IAV infection. This study investigated the cytokine/chemokine profiles associated with different diseases induced by IAV. Influenza A/WSN/33 (H1N1) virus, which has the ability to infect multiple organs, was used throughout this study to avoid disparities between IAV strains. We generated pneumonia and encephalitis mouse models by intranasal and intracranial inoculation with a lethal dose of IAV, respectively, to investigate the effects of infection site on cytokine/chemokine profiles. Results revealed no detectable virus in the brain in the pneumonia model or in the lung in the encephalitis model.

As a result of IAV infection, the concentrations of almost all examined cytokines and chemokines were significantly increased both in serum and in local samples (BALF or CSF) from the site of infection. Several reports of clinical studies have described systemic and local levels of cytokines and chemokines in patients with IAV infection of various types [8–11], but data related to the correlation between different types of diseases (e.g., pneumonia versus encephalitis) or samples (e.g., serum versus BALF) are scarce. In this study, we found the cytokine/chemokine profiles in serum and local samples (BALF in pneumonia or CSF in encephalitis) to compare the systemic and local immune responses both in pneumonia and in encephalitis models induced by IAV infection.

This study revealed visually, with radar charts, that the local cytokine/chemokine profiles differed depending on the infection site, although the serum profiles were similar. The shift to the lower area in the radar chart for CSF contributed to the difference between local cytokine/chemokine profiles. This finding indicated the more predominance of Th2 cytokines (IL-10, IL-13) in CSF than in BALF following IAV infection. These cytokines are classified as anti-inflammatory cytokines, which are fundamentally important for ameliorating inflammatory response and thereby preventing excessive host damage. Earlier reports described that IL-13 induces cell death of activated microglia, which is important for the prevention of chronic inflammation [16]. Elevated IL-10 concentrations in CSF are also reportedly associated with mild encephalitis/encephalopathy during IAV infection with a reversible splenial lesion with a good clinical course, which indicates that IL-10 might work to localize the lesion and to prevent sequelae [17]. Puntambekar et al. recently reported that IL-10 limited the expansion of CNS damage following viral-induced demyelination [18]. These unique findings related to the local immune response in the brain might be associated with characteristics of immune privilege [19]. However, further studies must be conducted to elucidate the cytokine/chemokine production mechanism.

Another key finding of this study was that the cytokine/chemokine profiles differed between serum and local samples (BALF or CSF) for the pneumonia and encephalitis models. Several reports have made specific mention of the difference of cytokine profiles between systemic and local samples in case series of influenza virus infection [9, 11, 20–24]. Some of them further presented the conclusion that the local samples are superior to serum samples for evaluating disease severity (Tables 1 and 2). These findings support our view of the importance of investigating local immune responses in IAV pathology.

Evaluating blood samples alone might be insufficient to understand the local immune reactions at the primary infection site. Therefore, using local samples from the infection site might be highly beneficial for elucidation of the clinical pathology of a particular patient and for devising an effective treatment plan. The reasons for discrepancies in cytokine and chemokine levels among sera and local samples were not explored in this study, but the topic might form the basis for further investigation of the innate immune responses during severe influenza virus infection (such as pneumonia and encephalitis) and therapeutic approaches. Whether specific cytokine and chemokine inhibitors have potential for severe influenza virus infection should be evaluated in future studies using appropriate experimental models.

## 5. Conclusion

In conclusion, local immune responses against IAV infection appear to vary depending on the infection site, whereas systemic immune responses remain almost similar. In addition, the cytokine and chemokine profiles in local immune responses might differ from those of systemic immune responses. Local samples such as CSF or BALF, which reflect the infection site pathology, are important for evaluating local immune responses and for aiding clinicians in devising the most appropriate treatment strategies.

## Figures and Tables

**Table 1:** Serum and BALF cytokine/chemokine profiles of patients affected influenza pneumonia among children, adolescents, and young adults.

Study	Virus type	Number of patients		Elevated cytokines and chemokines	
		Serum	BALF	Serum	BALF
Arankalle et al.	H1N1 2009	15	15	IL-1*β*, IL-6, IL-12p40, TNF-*α*, IL-10, MIP-1*α*	IL-12p40 was higher than serum. Remaining items were same levels as serum.
Zuniga et al.	H1N1 2009	42	42	IFN-*γ*, IL-6, MCP-1	IL-6, MCP-1

BALF: bronchoalveolar lavage fluid; IFN: interferon; IL: interleukin; MCP: macrophage chemotactic factor; MIP: macrophage inflammatory protein; TNF: tumor necrosis factor.

**Table 2:** Serum and CSF cytokine/chemokine profiles of patients affected influenza associated encephalopathy among children, adolescents, and young adults.

Study	Virus type	Number of patients		Elevated cytokines and chemokines	
		Serum	CSF	Serum	CSF
Aiba et al.	H3N2	6	2	IL-6, TNF-*α*, sTNF-R1	IL-6
Ichiyama et al.	H1N1, H3N2, B	14	10	IL-6, sTNF-R1, IL-10	IL-6, sTNF-R1
Hosoya et al.	H3N2, A, B	10	8	TNF-*α*	None
Hasegawa et al.	H1N1 2009	6	4	IFN-*γ*, IL-6, sTNF-R1, IL-10	IL-6
Momonaka et al.	H1N1 2009	18	18	IL-6	IL-6

CSF: cerebrospinal fluid; IFN: interferon; IL: interleukin; sTNF-R: soluble tumor necrosis factor receptor; TNF: tumor necrosis factor.
